# Characteristics and clinical outcomes of pediatric patients following massive bowel resection: A retrospective cohort study

**DOI:** 10.1016/j.intf.2024.100008

**Published:** 2024-07-16

**Authors:** Clarelle L. Gonsalves, Christina Belza, Glenda Courtney-Martin, Yaron Avitzur, Jill Quirt, Victoria Srbely, Paul W. Wales

**Affiliations:** a Group for Improvement of Intestinal Function and Treatment (GIFT), The Hospital for Sick Children, Toronto, Canada; b Transplant and Regenerative Medicine Center, The Hospital for Sick Children, Toronto, Canada; c Research Institute, The Hospital for Sick Children, Toronto, Canada; d Department of Nutritional Sciences, University of Toronto, Toronto, Canada; e Division of Gastroenterology, Hepatology and Nutrition, Department of Paediatrics, University of Toronto, Toronto, Canada; f Cincinnati Center of Excellence in Intestinal Rehabilitation (CinCEIR), Cincinnati Children’s Hospital Medical Center, Cincinnati, USA; g Division of General and Thoracic Surgery, Department of Surgery, Cincinnati Children’s Hospital Medical Center, University of Cincinnati, Cincinnati, USA

**Keywords:** Intestinal failure, Short bowel syndrome, Pediatrics, Parenteral nutrition

## Abstract

**Background:**

Massive bowel resection (MBR)**,** defined as residual small bowel < 25 % of expected length-for-age based on established norms**,** is a rare entity. Traditionally, children who have undergone MBR have higher mortality and less probability of achieving enteral autonomy compared to patients with less substantial bowel loss. The primary objective of this study was to determine the characteristics and clinical outcomes of pediatric patients following MBR. Secondary objectives included comparing patients who achieved enteral autonomy to those who remained parenteral nutrition (PN)-dependent to determine characteristics associated with PN independence.

**Methods:**

Retrospective cohort study of patients following MBR managed by our multidisciplinary intestinal rehabilitation program between January 1, 2006 and December 31, 2017, with an observation period ending December 31, 2021.

**Results:**

61 patients with MBR fulfilled study inclusion criteria. 21 patients (34.4 %) achieved enteral autonomy while 40 patients (65.6 %) did not. Patients who achieved enteral autonomy had a greater percentage of residual small bowel (22 % vs 13 %, P < 0.01) and colon (100 % vs 50 %, P < 0.01). They were more likely to have an ICV (73.2 % vs 15 %, P < 0.01) and less likely to undergo STEP procedure (14.3 % vs 37.5 %, P = 0.02). 18 patients in the cohort without enteral autonomy achieved greater than 50 % enteral nutrition. In this cohort, the overall transplant rate was 9.8 % and mortality rate was 11.5 %.

**Conclusion:**

Several important advancements have occurred over the last two decades that have allowed more patients to reach their adaptive potential and mitigate complications of prolonged PN following MBR. Overall outcomes are better than previously reported.

WHAT IS KNOWN

• Children who have undergone massive bowel resection have higher mortality and lower chance of achieving enteral autonomy compared with those with less substantial bowel loss
• Outcomes of this population are associated with several factors including residual bowel anatomy, number of sepsis events, and care by a multidisciplinary team, the ultimate predicator of mortality is failure to reach enteral autonomy

WHAT IS NEW

• This study found no significant differences in gestational age, birth weight, or etiology leading to MBR between patients who did and did not achieve enteral autonomy
• This study showed that enteral autonomy is possible for patients identified as having ultrashort bowel syndrome (USBS) and overall mortality is similar to those with more residual bowel
• Mortality has significantly decreased and life expectancy has increased in patients with residual bowel length < 25% of that predicted for age

## Introduction

Pediatric intestinal failure (PIF) is a complex and devastating medical condition that is characterized as the inability of the gastrointestinal tract to absorb adequate nutrition and fluids to sustain life. [1] It has been defined as the reduction of functional intestinal mass below that which can sustain life, resulting in dependence on supplemental parenteral support for a minimum of 60 days within a 74 consecutive day interval. [2] Intestinal failure results from a variety of alterations of normal anatomy and physiology and is often further compounded by a constellation of nutritional, infectious and metabolic complications. [3] Pediatric IF is caused by short bowel syndrome (SBS), dysmotility disorders and mucosal enteropathies. [3], [4], [5], [6] The most common cause of PIF is short bowel syndrome (SBS) with an estimated incidence of 24.5 per 100,000 live births. [7] Pediatric SBS is typically caused by congenital anomalies of the gastrointestinal (GI) tract or acquired conditions of the newborn such as necrotizing enterocolitis. Historical mortality estimates have been as high as 35–50 %, however, more recent findings show mortality of ranging from 10.5 %− 27 % for patients diagnosed between 2000 and 2015. [7], [8], [9], [10], [11], [12].

Children having undergone massive bowel resection (MBR) is a rarer event. In order to accurately characterize pediatric massive bowel resection, reference intestinal lengths for post-conceptual age, weight, and length at time of surgery have been developed. [13] The specific incidence and mortality following massive bowel resection in pediatric patients is not known. However, there is a trend towards increasing numbers due to increased incidence of short bowel syndrome and improved survival amongst patients with increasingly limited bowel length. Irrespective of this, greater residual small bowel length has consistently been associated with improved survival and rates of enteral autonomy in children with SBS. [14], [15], [16].

Children who have undergone massive bowel resection have higher mortality and lower chance of achieving enteral autonomy compared with those with less substantial bowel loss. [17] Until recently, MBR in infants has been considered an indication for intestinal transplantation or termination of life support measures as the prognosis was considered futile. However, with advancements in operative techniques, the establishment of multidisciplinary intestinal rehabilitation programs, and efforts for early referral to intestinal transplantation, there has been improvement in outcomes for these patients. Although it is known that outcomes of this population are associated with several factors including residual bowel anatomy, number of sepsis events, and care by a multidisciplinary team, the ultimate predicator of mortality is failure to reach enteral autonomy. [17] Limited current understanding of the proportion of children expected to reach enteral autonomy following extensive gut loss has implications on early decisions regarding resuscitation, surgical and medical management of resultant IF. [18] There is, however, a paucity of studies examining the characteristics and outcomes of the small subpopulation of patients following MBR. The available studies are limited by small sample size, patient heterogeneity and the lack of long-term follow up.

The primary objective of this study was to determine the characteristics and clinical outcomes of pediatric patients who have undergone MBR. We also compared patients who achieved enteral autonomy to those who remained dependent on parenteral nutrition to determine characteristics associated with PN independence.

## Methods

We performed a retrospective cohort study of all patients managed by our multidisciplinary intestinal rehabilitation program who underwent massive bowel resection. MBR was defined as a residual bowel length < 25 % of that predicted for age based on established norms. [13] Patients referred to our program between January 1, 2006 through December 31, 2017 were enrolled. Study observation period continued through December 31, 2021 to ensure a minimum 4 year follow up period. Patients were excluded if they had a severe, life-threatening anomaly (e.g., cardiac), chromosomal abnormalities/syndromes (trisomies 13 and 18), or comorbid conditions (grade III/IV intraventricular hemorrhage, chronic lung disease) that would preclude surgical management.

Data collection included patient demographics, surgical interventions, intestinal anatomy, and outcomes (nutrition, sepsis, intestinal failure associated liver disease, central venous catheter complications, transplantation, and mortality). Data on length of parenteral nutrition therapy, ability to reach 50 % and complete enteral autonomy, as well as length of follow up were also collected. Enteral autonomy was defined as independence from all parenteral support with maintenance of hydration and growth for minimum 12 weeks. Cholestasis and advanced cholestasis were defined as serum conjugated bilirubin > 50umol/L and > 100umol/L, respectively sustained for > 2weeks and not associated with a septic event.

Descriptive statistics were used to summarize the cohort employing medians with interquartile ranges for continuous variables and frequencies with proportions for categorical variables. Analysis was performed both for the entire cohort, as well as, stratified by patients who had and had not reached enteral autonomy by the end of the study observation period. Sub-group analysis was done on patients within this cohort who met criteria for ultrashort bowel syndrome, defined as those with < 10 % residual small bowel length for age. [2].

Statistical analysis was completed using Mann Whitney U and Chi square test as appropriate. Multivariable logistic regression was performed with the dependent variable being achievement of enteral autonomy. Variables significant on the univariate analysis with a P-value < 0.2 were included into the model in a backward step-wise approach. Odds Ratios (OR) with 95 % confidence intervals were determined. An alpha value < 0.05 was considered to be significant.

## Results

A total of 61 patients who underwent MBR fulfilled the study’s inclusion criteria. Forty-two (69 %) of these patients were male. The median gestational age of this cohort was 35.0 weeks (32.0–38.0 weeks) with a median birthweight of 2.3 kg (1.8–2.8 kg). The most common etiologies of MBR were complicated gastroschisis in 23 patients (37.7 %) and necrotizing enterocolitis in 13 patients (21.3 %). Overall, 21 patients (34.4 %) achieved enteral autonomy by the end of the study period. There were no significant differences in patient characteristics between groups (Table 1).

**Table 1:** Patient Characteristics.

	**Entire Cohort (n = 61)**	**Enteral autonomy (n = 21)**	**No enteral autonomy** **(n = 40)**	**p-value**
Median (IQR)/Frequency (%)				
Male (%)	42 (69.0)	14 (66.7)	28 (69.2)	0.84
Gestational age (weeks)	35.(32.0-38.0)	35.0 (34.0-37.0)	34.0 (32.0-38.0)	0.72
Birth weight (kg)	2.3 (1.8-2.8)	2.3 (1.9-2.8)	2.3 (1.8-2.8)	0.76
Etiology				0.52
NEC	13 (21.3)	6 (28.6)	7 (17.5)	
Atresia	9 (14.8)	4 (19.0)	5 (12.5)	
Gastroschisis	23 (37.7)	5 (23.8)	18 (45.0)	
Volvulus	8 (13.1)	3 (14.3)	5 (12.5)	
Hirschsprung	3 (4.9)	0 (0.0)	3 (7.5)	
Meconium ileus	1 (1.6)	1 (4.8)	0 (0.0)	
Other	4 (6.6)	2 (9.5)	2 (5.0)	
NEC vs other diagnoses	13 (21.3)	6 (28.6)	7 (17.5)	0.48
Inflammatory etiology versus other diagnoses*	37 (60.7)	12 (57.1)	25 (62.5)	0.74
Gastroschisis vs other diagnoses	23 (37.7)	5 (23.8)	18 (45.0)	0.18

NEC=necrotizing enterocolitis

*Inflammatory etiologies included necrotizing enterocolitis, gastroschisis, and meconium peritonitis

The median residual small bowel length for the entire cohort was 22.0 cm (15.0–35.0 cm) which represented 15.0 % (10.0–22.0 %) of expected small bowel length corrected for age. [13] Median residual large bowel length was 30.0 cm (19.0–38.0 cm) representing 75.0 % (45.0–100.0 %) of expected colon length for age. A total of 39 patients (63.9 %) had lost their ileocecal valve (ICV) and 18 (29.5 %) underwent a serial transverse enteroplasty (STEP) procedure (Table 2). There were significant differences in residual bowel anatomy between patients who achieved enteral adaptation compared to those who did not. Remnant small bowel length was 30.0 cm (22.0–40.0 cm) for the enteral autonomy group and 18.0 cm (14.0–30.0 cm) for the PN dependent group (P < 0.01). The percent residual small bowel adjusted for age was 22.0 % (14.5–24.0 %) and 13.0 % (7.5–18.9 %), respectively (P < 0.01). The median percentage of expected large bowel remaining was 100 % (100.0–100.0 %) in the enteral autonomy group versus 50.0 % (38.3–82.5 %) (P < 0.01) for the group without enteral autonomy (P < 0.01). Finally, significant differences were found in the number of patients who had an ICV resected [5 (28.6 %) versus 34 (85.0 %), (P < 0.01)], as well as, in the number of patients who underwent a STEP procedure [3 patients (14.3 %) who achieved enteral autonomy versus 18 patients (37.5 %) in the PN dependent group (P = 0.02)] (Table 2).

**Table 2:** Residual Intestinal Anatomy.

	**Entire cohort (n = 61)**	**Enteralautonomy** **(n = 21)**	**No enteral autonomy** **(n = 40)**	**p-value**
Median (IQR)/Frequency (%)				
Small bowel length (cm)	22.0 (15.0-35.0)	30.0 (22.0-40.0)	18.0 (14.0-30.0)	**< 0.01**
% Small bowel remaining	15.0 (10.0-22.0)	22.0 (14.5-24.0)	13.0 (7.5-18.9)	**< 0.01**
Large bowel length (cm)	30.0 (19.0-38.0)	30.0 (26.0-40.0)	25.0 (15.8-35.0)	**0.03**
% Large bowel remaining	75.0 (45.0-100.0)	100.0 (100.0-100.0)	50.0 (38.8-82.5)	**< 0.001**
ICV resected (%)	39 (63.9)	5 (28.6)	34 (85.0)	**< 0.001**
Stoma (%)	37 (60.7)	12 (57.1)	25 (62.5)	0.74
Colostomy (yes)	18 (29.5)	4 (19.0)	14 (35.0)	0.24
Initial Stoma location (%)				
Jejunostomy	25 (41.0)	11 (52.4)	14 (35.0)	0.48
Ileostomy	3 (5.3)	0 (0.0)	3 (7.5)	
Colostomy	6 (10.5)	1 (4.8)	5 (12.5)	
STEP procedure (yes)	18 (29.5)	3 (14.3)	15 (37.5)	0.14

ICV=ileocecal valve

STEP procedure= serial transverse enteroplasty

Table 3 summarizes other clinical outcomes. The median number of days on PN was 219 (171.5–491.8) days for the enteral autonomy group and 2013 (728.0–2822.0) days for those who did not achieve enteral autonomy. Additionally, 18/41 (43.9 %) patients in the PN dependency group were able to achieve greater than 50 % enteral calories. Of the entire cohort, 46/61 (75.0 %) experienced at least one septic event, with a rate of 1.1 (0.1–4.6) CLABSI event per 1000 CVC days and 4.8 (1.9–8.3) CVC complications (breakage, dislodgement, and occlusion) per 1000 CVC days. Twenty-seven patients (44.3 %) in the entire population developed cholestasis. There was no significant difference in the development of cholestasis between groups or the median days to cholestasis between groups. Advanced cholestasis occurred in 12/61 (19.7 %) patients. Although the incidence of advanced cholestasis was not significant between groups, there was a significant difference in the median days to advanced cholestasis [98.0 (93.0–141.0)] in the enteral adaptation group versus 234.0 (178.0–442.0) in the group who did not achieve enteral adaptation.

**Table 3:** Clinical Outcomes.

	**Total cohort (n = 61)**	**Enteralautonomy** **(n = 21)**	**No enteral autonomy (n = 40)**	**p-value**
Median (IQR)/Frequency (%)				
Central Line Complications				
*CLABSI (%)	46 (75.0)	14 (66.7)	32 (80.0)	**0.01**
CLABSI/1000 CVC days	1.1 (0.1-4.6)	1.2 (0-6.7)	1.2 (0.3-4.2)	0.97
^#^CVC complication/1000 CVC days	4.8 (1.9-8.3)	8.9 (4.8-12.1)	3.6 (1.7-6.1)	**< 0.001**
Nutrition				
PN days	1444 (426-3218)	219 (171-491)	2013 (728-2822)	
Achieved > 50 % enteral nutrition (%)	37 (60.7)	21 (100.0)	18 (45.0)	**< 0.001**
Liver				
Cholestasis (34umol/L) (%)	27 (44.3)	8 (38.1)	17 (42.5)	0.98
Days to cholestasis	80 (50-125)	65 (58-98)	124 (80-192)	0.38
Advanced IFALD (100umol/L) (%)	12 (19.7)	5 (23.8)	8 (20.0)	0.70
Days to Advanced IFALD (100 ug/L)	136 (102-216)	98 (93-141)	234 (178-442)	**0.04**
Transplant (%)	6 (9.8)	1 (4.8)	5 (12.5)	0.62
Liver	1 (1.6)	0 (0.0)	1 (2.5)	
Liver + Intestinal	1 (1.6)	1 (4.8)	0 (0.0)	
Multi-visceral	4 (6.6)	0 (0.0)	4 (10.0)	
Follow-up				
Hospitalization (days)	110 (36-173)	141 (92-177)	74 (32-157)	0.22
Mortality	7 (11.5)	2 (9.5)	5 (12.5)	0.86
Length of follow up	1673 (729-3270)	1093 (663-3161)	1969 (995-2816)	0.84

*CLABSI: number of patients who experienced a minimum of one central line associated bloodstream infection event

CVC=central venous catheter; ^#^CVC complications = breakage, dislodgement, occlusion

PN=Parenteral Nutrition; IFALD=Intestinal failure associated liver disease

* * Fisher’s exact test

A total of six patients (9.8 %) underwent transplantation [1 isolated liver, 1 combined liver/bowel transplant and 4 multi-visceral]. The indication for transplant in all six patients was liver failure. The mortality rate of the entire cohort, including post-transplant mortality, was 11.5 %, representing seven patients. Causes of death included respiratory (n = 1), central nervous system (n = 2), multisystem (n = 2) and other (n = 1). There was one incidence of post-transplant mortality in this cohort which was secondary to post-transplant lymphoproliferative disorder (PTLD) and associated hemophagocytic lymphohistiocytosis (HLH). Multivariable analysis in the form of backward stepwise regression found significant predictors of enteral autonomy to be residual small bowel percentage and large bowel percentage (Table 4).

**Table 4:** Multivariable analysis evaluating predictors of enteral autonomy (R^2^ =0.52).

**Predictor**	**Adjusted beta coefficient (standard error)**	**OR**	**95 % CI**	**p-value**
Percent Small bowel expected for age	0.149 (0.060)	1.160	1.031-1.306	**0.014**
Percent Large bowel expected for age	0.028 (0.014)	1.029	1.001-1.057	**0.043**

A total of 16 patients in this cohort were identified as those with ultrashort bowel syndrome (USBS), having < 10 % residual small bowel length for age. The median residual percentage of expected small bowel for age in this group was 5.0 %. Gastroschisis was the most common etiology resulting in bowel loss in this subgroup (43.8 %). Of these patients, two (12.5 %) achieved enteral autonomy while 14 (87.5 %) remained PN dependent at the end of the observation period. Of this group, 50.0 % achieved > 50 % enteral autonomy. Mortality rate in this subgroup was 12.5 % (Table 5**)**.

**Table 5:** Patient characteristics, residual intestinal anatomy, and clinical outcomes for patients with Ultrashort bowel syndrome (<10 % residual small bowel for age).

	**Total** **(n = 16)**	**Enteral** **autonomy** **(n = 2)**	**No enteral autonomy (n = 14)**
Median (IQR)/Frequency (%)			
**Patient Characteristics**			
Male (%)	11 (68.8)	1 (50.0)	10 (71.4)
Gestational age (weeks)	36.0 (35.0-38.0)	38.0 (37.0-39.0)	36.0 (35.0-40.0)
Birth weight (kg)	2.51 (2.14-2.82)	2.95 (2.5-3.4)	2.5 (2.2-3.5)
Etiology			
NEC	0 (0.0)	0 (0.0)	0 (0.0)
Atresia	2 (12.5)	0 (0.0)	2 (14.3)
Gastroschisis	7 (43.8)	1 (50.0)	6 (42.9)
Volvulus	4 (25.0)	1 (50.0)	3 (21.4)
Hirschsprung	1 (6.2)	0 (0.0)	1 (7.1)
Meconium ileus	0 (0.0)	0 (0.0)	0 (0.0)
Other	2 (12.5)	0 (0.0)	2 (14.3)
NEC vs other diagnoses	0 (0.0)	0 (0.0)	0 (0.0)
Inflammatory etiology versus other diagnoses*	7 (43.8)	1 (50.0)	6 (42.9)
Gastroschisis vs other diagnoses	7 (43.8)	1 (50.0)	6 (42.9)
**Residual Intestinal Anatomy**			
Small bowel length (cm)	12.0 (4.1-14.0)	7.0 (5.0-9.0)	13.0 (4.9-14.0)
% Small bowel remaining	5.0 (3.0-8.0)	4.0 (3.5-4.5)	5.0 (3.0-8.0)
Large bowel length (cm)	30.0 (16.5-40.0)	28.0 (21.25-33.75)	30.0 (17.5-40.0)
% Large bowel remaining	75.0 (50.0-100.00)	85.0 (82.5-87.5)	63.0 (50.0-100.0)
ICV resected (%)	12 (75.0)	2 (100.0)	10 (71.4)
Stoma (%)	10 (62.5)	1 (50.0)	9 (64.3)
Colostomy (yes)	8 (50.0)	1 (50.0)	7 (50.0)
Initial Stoma location (%)			
Jejunostomy	4 (25.0)	0 (0.0)	4 (28.6)
Ileostomy	1 (6.2)	0 (0.0)	1 (7.1)
Colostomy	2 (12.5)	1 (50.0)	1 (7.1)
STEP procedure (yes)	5 (31.3)	0 (0.0)	5 (35.7)
**Clinical Outcomes**			
Central Line Complications			
*CLABSI (%)	13 (81.3)	2 (100.0)	11 (78.6)
CLABSI/1000 CVC days	1.13 (0.25-2.17)	2.35 (1.3-3.4)	1.13 (0.37-2.11)
^#^CVC complication/1000 CVC days	3.57 (3.03-4.44)	3.5 (3.0-4.0)	3.57 (3.1-4.9)
Nutrition			
PN days	2616 (1264-3914)	896 (680-1112)	2615 (1532-3900)
Achieved > 50 % enteral nutrition (%)	8 (50.0)	2 (100.0)	6 (42.9)
Liver			
Cholestasis (34umol/L) (%)	7 (43.8)	1 (50.0)	6 (42.9)
Days to cholestasis	87 (50-199)	87 (87-87)	93 (47-233)
Advanced IFALD (100umol/L) (%)	3 (18.8)	1 (50.0)	2 (14.3)
Days to Advanced IFALD (100 ug/L)	140 (126-372)	113 (113-113)	372 (256-488)
Transplant (%)	1 (6.3)	0 (0.0)	1 (7.1)
Liver	0 (0.0)	0 (0.0)	0 (0.0)
Liver + Intestinal	0 (0.0)	0 (0.0)	0 (0.0)
Multi-visceral	1 (6.3)	0 (0.0)	1 (7.1)
Follow-up			
Hospitalization (days)	82 (38-149)	28 (18-37)	101 (45-141)
Mortality	2 (12.5)	0 (0.0)	2 (14.3)
Length of follow up (days)	2947 (1350-3913)	2052 (1115-2987)	2947 (1518-3898)

*Inflammatory etiologies included necrotizing enterocolitis, gastroschisis, and meconium peritonitis

* *CLABSI: number of patients who experienced a minimum of one central line associated bloodstream infection event

NEC=necrotizing enterocolitis, ICV=ileocecal valve, STEP=serial transverse enteroplasty, CVC=central venous catheter; ^#^CVC complications = breakage, dislodgement, occlusion

PN=Parenteral Nutrition; IFALD=Intestinal failure associated liver disease

## Discussion

Children who have undergone MBR represent an important subgroup of children with SBS. Although it is historically known that children with residual bowel length < 25 % of that predicted for age have higher mortality and a reduced probability of achieving enteral autonomy when compared to those with greater residual bowel length, this remains an understudied population. This is particularly true in the context of improvements realized in the current era of the management for children with intestinal failure over recent years. As a result, our study aimed to characterize the clinical outcomes of pediatric patients following MBR to determine whether there were any characteristics associated with those who achieved enteral autonomy.

This study included 61 patients with less than 25 % residual small bowel length of that predicted for age with a follow up period of four years. We demonstrated that the overall survival of these complex patients was 89 %, The overall transplant rate in this study population was 9.8 %. The incidence of enteral autonomy was 34.4 % and of patients remaining PN-dependent, 18/40 (45.0 %) tolerated at least 50 % of their nutrition enterally. We found no significant differences in gestational age, birth weight, or etiology leading to MBR between patients who did and did not achieve enteral autonomy. Furthermore, a subset of these patients identified as having ultrashort bowel syndrome (USBS), defined as < 10 % residual small bowel length for age, revealed that enteral autonomy is possible in this subgroup of patients and overall mortality is similar to those with more residual bowel. Until the recent update of intestinal listing criteria [(1) ≥ 2 intensive care unit admissions requiring intubation or inotropic support, (2) persistent elevation of conjugated bilirubin **≥** 75 mmol/L following 8 weeks of lipid management strategies, (3) loss of **≥** 3 central venous catheter sites out of the upper and lower body central venous catheter sites] [19], MBR had been considered an indication for intestinal transplantation. The findings of this study provide further evidence to illustrate that prior listing criteria that included MBR were not reflective of the current era of management, and for individuals in whom transplantation was previously believed to be necessary, they have excellent long-term survival and many are able to achieve enteral autonomy and PN reduction. Despite some limited literature acknowledging improved outcomes for pediatric patients following MBR, a recent study by Cairns et al. suggests that this has not yet translated to a widespread and consistent change in practice and counselling for families of children who require massive bowel resection, primarily amongst surgeons and neonatologists who are integral to the early care of these children - many of whom continue to believe that massive bowel resection is a palliative diagnosis. [20] Our findings highlight that overall outcomes are better than previously reported and thus serve to further emphasize that counselling families at time of massive bowel resection should be reflective of this.

It is not surprising that enteral autonomy was associated with a longer residual small intestine and preservation of the ICV, which is a proxy for preservation of the terminal ileum. As we have shown in a previous publication, a longer colon remnant in continuity is positively associated with enteral autonomy when the small bowel remnant is < 50 % of that expected for age. [21] STEP procedures were more common in children who did not achieve enteral autonomy. This reflects the pattern of practice at our institution where STEP procedures tend to be performed in patients with more extreme anatomy.

Several important advancements in management of PIF have occurred over the last two decades that have allowed more patients with SBS to reach their adaptive potential and mitigate complications of prolonged PN. The first has been the establishment of multidisciplinary intestinal rehabilitation teams as described above. Until the most recent definition of USBS^2^, definitions used in the literature to describe this patient population have varied overtime, ranging from < 40 cm to < 10 cm of residual small bowel, and therefore, are representative of patients with MBR. Although more recent literature supports improved survival and decreased morbidity and mortality in patients following MBR [22], a 2017 study including patients with residual small bowel length of less than 10 cm by Dore et al. reported a mortality rate of 27 %. [23].

Historically, IF-associated liver disease (IFALD) was the major cause of mortality in children with IF. [7], [11] Several studies have reported estimates of 30–90 % of patients developing some degree of cholestasis, with a smaller percentage progressing to end stage liver disease. [9], [24], [25], [26], [22], [23], [27] Our study found that 44.3 % of the entire cohort of children with MBR developed some degree of cholestasis that was typically reversible. The availability of composite lipid emulsions such as SMOFlipid in this population has been associated with lower incidence and decreased severity of IFALD, thus preventing or at the very least delaying onset of liver complications and therefore increasing time for adaptation. [28] SMOFlipid has been our default lipid emulsion at our institution since 2013. Reduction of septic complications with introduction of ethanol locks [29] and more recently 4 % Tetrasodium-EDTA [30] has reduced the incidence of IFALD and has also been associated with greater gut adaptation. [19] We adopted a more aggressive approach to autologous bowel reconstruction and implementation/introduction of enteral nutrition several years ago that we believe has also contributed to reduced morbidity [31].

Improvement in medical and surgical management has resulted in fewer patients being listed for transplantation. [32] Since 2006, 9.8 % of this more complex subgroup of patients with MBR received transplantation. This is less than the rate of transplantation reported by the two multi-institutional studies of PIF patients by Squires et al. in 2012 (25 %) and Gattini Valdes et al. in 2021 (17 %). [12], [33] Overall, survival was 89 %. No patients died of IF related causes such as IFALD or sepsis. This reflects the improvement in outcomes in the modern era of management. In fact, most mortality is now related to comorbidities related to prematurity. [34].

The available outcome data in the literature for MBR is sparse. This cohort represents a total of 15 years of intestinal rehabilitation experience with a minimum of four years of follow up per subject. To the best of our knowledge, this study represents the longest follow up reported, and this data illustrates the advancements in the PIF treatment over the last two decades. With respect to future directions for this population, the recent approval of Teduglutide, an analogue of Glucagon Like Peptide-2 (GLP-2) for the promotion of intestinal adaptation, may further increase the proportion of children with USBS who go on to achieve enteral autonomy in the years to come. With the introduction of novel therapeutic modalities such as trophic peptides it is important to understand where practice and outcomes currently stand, at baseline in patients with extreme anatomy and have this reflected in the literature prior to the widespread availability of these new therapeutic modalities to permit a more meaningful understanding of future outcomes.

We acknowledge that this study is not without limitations. The sample size is small precluding our ability to perform more in-depth modelling to explore associations with enteral autonomy. Additionally, this study excluded patients with severe life-threatening anomalies such as cardiac conditions, chromosomal abnormalities/syndromes, and comorbid conditions (grade III/IV intraventricular hemorrhage, chronic lung disease) that would preclude surgical management. As a result, these results do not capture morbidity and mortality for all children following massive bowel resection.

To date, our understanding of risk factors, extraintestinal factors and prognosis for children following MBR is based on limited small case series and studies, with limited long-term follow up intervals. In the current era of management for PIF, mortality has decreased significantly and patients with residual bowel length < 25 % of that predicted for age are surviving longer.

## FUNDING

This research did not receive any specific grant from funding agencies in the public, commercial or not-for-profit sectors.

## Ethical Statement

Ethics approval has been obtained per local ethical board process with their letter number 1000066761.

## CRediT authorship contribution statement

**Glenda Courtney-Martin:** Writing – review & editing, Conceptualization. **Yaron Avitzur:** Writing – review & editing, Supervision, Project administration, Methodology, Data curation, Conceptualization. **Jill Quirt:** Writing – review & editing, Project administration, Formal analysis, Data curation. **Victoria Srbely:** Writing – review & editing, Project administration. **Paul W Wales:** Writing – review & editing, Writing – original draft, Supervision, Methodology, Investigation, Formal analysis, Data curation, Conceptualization. **Clarelle L Gonsalves:** Writing – review & editing, Writing – original draft, Conceptualization. **Christina Belza:** Writing – review & editing, Writing – original draft, Supervision, Project administration, Methodology, Investigation, Formal analysis, Data curation, Conceptualization.

## Declaration of Competing Interest

The authors declare the following financial interests/personal relationships which may be considered as potential competing interests: Paul Wales reports a relationship with Baxter that includes: funding grants. Paul Wales reports a relationship with Takeda that includes: funding grants. If there are other authors, they declare that they have no known competing financial interests or personal relationships that could have appeared to influence the work reported in this paper.
